# Cleaner shrimp are a sustainable option to treat parasitic disease in farmed fish

**DOI:** 10.1038/s41598-018-32293-6

**Published:** 2018-09-18

**Authors:** David B. Vaughan, Alexandra S. Grutter, Kate S. Hutson

**Affiliations:** 1 Centre for Sustainable Tropical Fisheries and Aquaculture, College of Science and Engineering, James Cook University, Townsville, Australia; 2 School of Biological Sciences, The University of Queensland, St Lucia, Queensland Australia

## Abstract

Chemical use is widespread in aquaculture to treat parasitic diseases in farmed fish. Cleaner fish biocontrols are increasingly used in fish farming as an alternative to medicines. However, cleaner fish are susceptible to some of their clients’ parasites and their supply is largely dependent on wild harvest. In comparison, cleaner shrimp are not susceptible to fish ectoparasites and they can be reliably bred in captivity. The effectiveness of shrimp in reducing parasites on farmed fish remained unexplored until now. We tested four cleaner shrimp species for their ability to reduce three harmful parasites (a monogenean fluke, a ciliate protozoan, and a leech) on a farmed grouper. All shrimp reduced parasites on fish and most reduced the free-living early-life environmental stages – a function not provided by cleaner fish. Cleaner shrimp are sustainable biocontrol candidates against parasites of farmed fish, with the peppermint cleaner shrimp reducing parasites by up to 98%.

## Introduction

Consumers are becoming increasingly aware of the impacts that their food choices have on the environment. In response, products that are produced sustainably communicate this sustainability through eco-labelling, which in turn informs client choice^1^. Farmed fish products are perceived as likely to contain higher amounts of antibiotics or other chemicals, and to be generally less healthy than wild-caught fish^2,3^. Thus, more naturally-produced farmed products are required.

Chemical therapy against parasitic disease remains commonplace in global aquaculture. There is considerable overlap in the antibiotics used in human medicines and animal food production, notably in aquaculture^4,5^, but other chemicals, such as organophosphates, avermectins, pyrethroids, and benzoylureas are also used^6^ with origins in agricultural pest control. Some of these chemicals may have negative environmental impacts^7^, but concerns have also been raised of direct resistance of fish parasites to some of these chemicals when used in aquaculture^6–11^.

Parasitic diseases (i.e., excluding viral and bacterial aetiologies) account for between 30% and 50% of annual stock losses in certain aquaculture industries in Asia^12^. Asia is the world’s largest aquaculture producing region^13,14^ of which Asian seabass (barramundi; *Lates calcarifer*) and groupers (*Epinephelus* spp.) contribute a significant proportion to the marine finfish sector^12,15^. In general, diseases influencing aquaculture are broadly categorised into three main groups: they potentially affect trade and are listed by the World Organisation for Animal Health (OIE), directly affect cultured species at various production levels, and are emergent diseases^14^. In Asian fish aquaculture, diseases arguably represent the latter two groups.

Of the many parasitic species recorded for marine finfish species in the Asia-Pacific region, the ciliate *Cryptocaryon irritans*, the monogenean *Neobenedenia girellae*, and the leech *Zeylanicobdella arugamensis* have economic significance, and are directly responsible for financial losses due to mortalities, or poor production performance^12^. Additionally, these parasites share similar life history strategies; each has a direct life cycle, and a diverse fish host range. The environmental stages (tomont cysts, eggs, or cocoons, respectively) are resistant to chemical therapies, resulting in eradication difficulties, imminent reinfections post fish treatment, and ongoing chronic-level disease. These characteristics, coupled with a broad tropical and sub-tropical geographic range^16–19^, contribute to the ectoparasites’ success in captive fish populations in this region, and elsewhere.

Employing biocontrols against parasites in aquaculture may reduce chemical intervention use. The Atlantic salmon aquaculture industry is the largest producer of cultured finfish in Europe^20^, and is currently the only aquaculture industry globally that employs native cleaner fish as biocontrols to effectively control ectoparasites^21–23^. Currently, no equivalent cleaner biocontrols are used commercially in sub-tropical or tropical aquaculture. Unfortunately, sub-tropical or tropical cleaner fishes would likely be susceptible to some of the ectoparasites they would be employed to control, especially those with a low host-specificity (e.g. *C. irritans*^24^, and some monogeneans^25^). Therefore, it is unlikely that a teleost cleaner biocontrol would be feasible for sub-tropical or tropical marine aquaculture. In contrast, cleaner shrimp, which are active predators of fish ectoparasites^26–30^, are not susceptible to the ectoparasites of fishes. However, they have never been directly evaluated as biocontrols in aquaculture.

Here, we investigated the ability of four cleaner shrimp species, *Lysmata amboinensis*, *Lysmata vittata*, *Stenopus hispidus*, and *Urocaridella antonbruunii* to reduce the parasites *C. irritans*, *N. girellae*, and/or *Z. arugamensis* (representing a protozoan, a monogenean, and a hirudinean fish ectoparasite) infesting the susceptible aquaculture host fish *Epinephelus coioides*, and the parasites’ respective tomont (cyst), egg, and cocoon environmental stage, under controlled laboratory conditions. We evaluated the ability of shrimp to perform diurnally and/or nocturnally, and provide for the first time an insight into different shrimp species’ preferences and abilities. Our results highlight and support the further investigation of *L. vittata* as the first cleaner shrimp biocontrol candidate for sub-tropical and tropical aquaculture.

## Results

**Figure 1:**
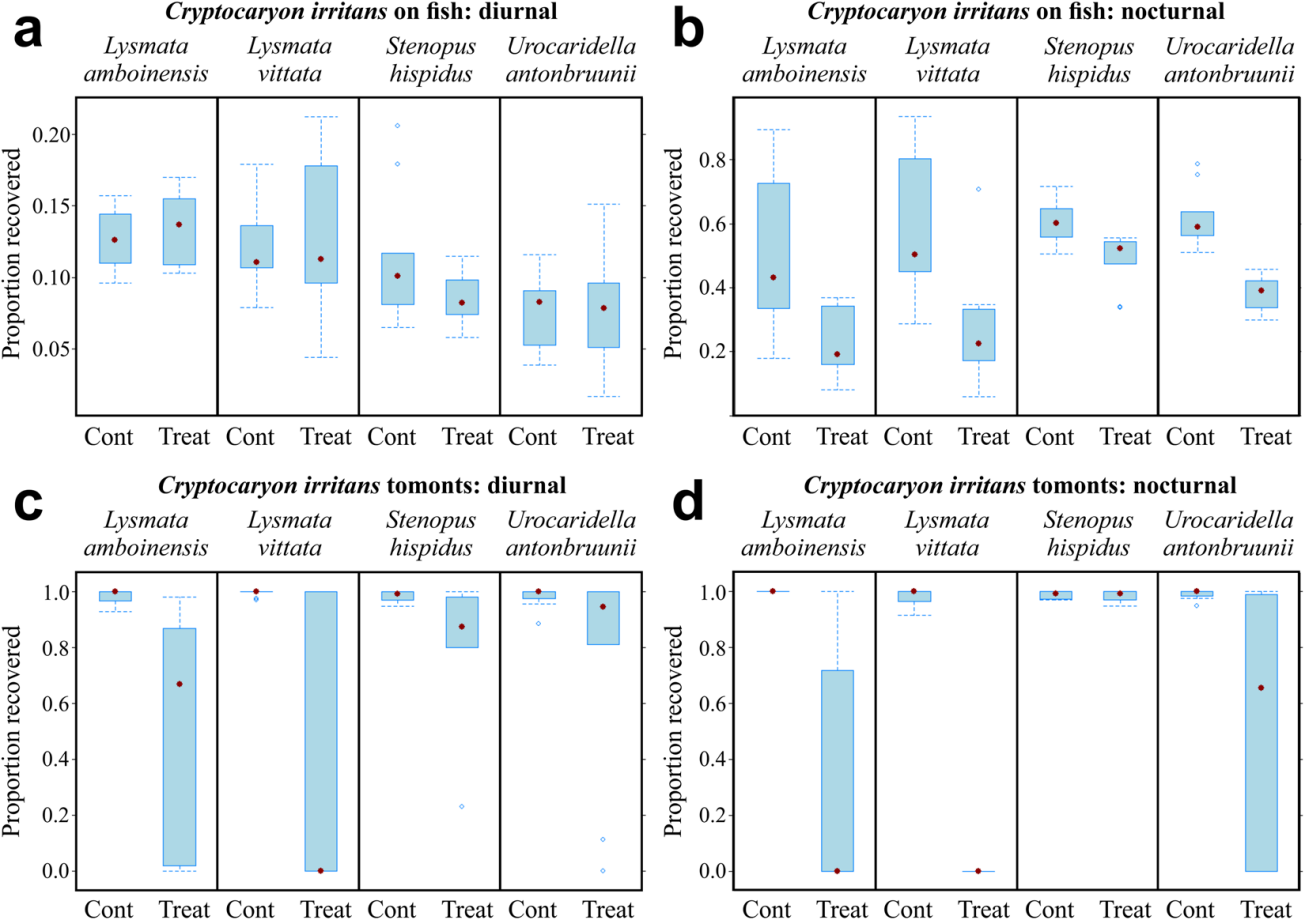
Effect of cleaner shrimp on *Cryptocaryon irritans*. (**a**) Reduction of *C. irritans* trophonts (parasitic stage) on fish by cleaner shrimp diurnally. (**b**) Nocturnally. (**c**) Reduction of *C. irritans* tomonts (environmental stage) diurnally. (**d**) Nocturnally. Data expressed as the proportion recovered.

**Figure 2:**
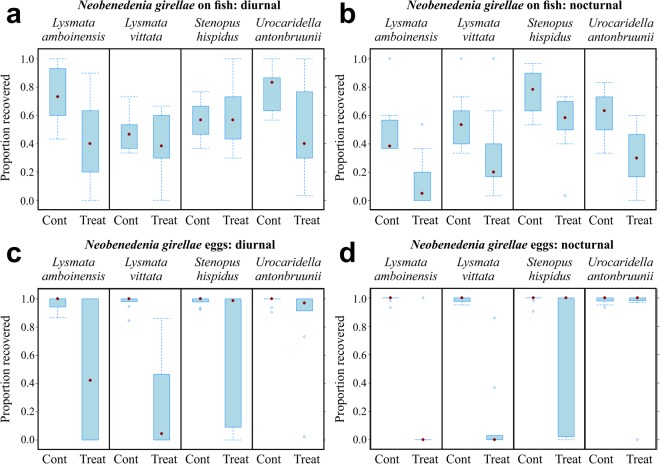
Effect of cleaner shrimp on *Neobenedenia girellae*. (**a**) Reduction of sub-adult *N. girellae* (parasitic stage) on fish by cleaner shrimp diurnally. (**b**) Nocturnally. (**c**) Reduction of *N. girellae* eggs (environmental stage) diurnally. (**d**) Nocturnally. Data expressed as the proportion recovered.

**Figure 3:**
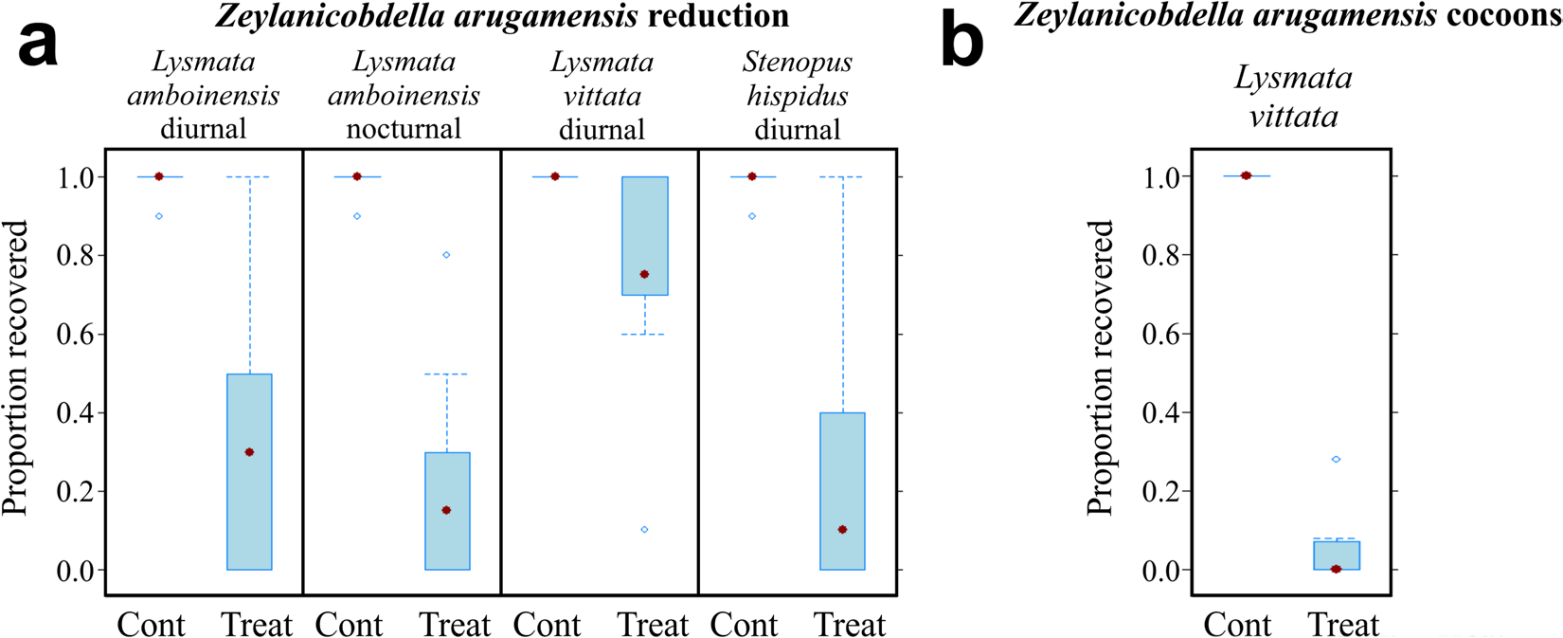
Effect of cleaner shrimp on *Zeylanicobdella arugamensis*. (**a**) Reduction of sub-adult *Z. arugamensis* (parasitic stage) by cleaner shrimp diurnally or nocturnally. (**b**) Reduction of *Z. arugamensis* cocoons (environmental stage) by *Lysmata vittata* over 24 hours. Data expressed as the proportion recovered.

Cleaner shrimp reduced parasite numbers on fish and their environmental stages under diurnal and nocturnal conditions (Figs 1–3), however shrimp species did not perform equally, and some species performed better at night. Two *L. vittata* were eaten by their clients during cohabitation at the completion of the nocturnal trials with *N. girellae* as the lights were turned on to recover the shrimp. This did not affect the result, and no other shrimp were eaten during the study.

A treatment effect was detected in the initial exploratory diurnal and nocturnal statistical models for the *C. irritans* environmental stage (tomonts: *χ*²_(1, *n* = 80)_ = 70.42, *p* < 0.001) and nocturnal model parasitic stage (on fish: *χ*²_(1, *n* = 80)_ = 176.91, *p* < 0.001; *χ*²_(1, *n* = 80)_ = 49.06, *p* < 0.001), both the diurnal and nocturnal models for the parasitic stage (on fish: *χ*²_(1, *n* = 80)_ = 48.99, *p* < 0.001; *χ*²_(1, *n* = 80)_ = 76.05, *p* < 0.001), and the environmental stage (eggs: *χ*²_(1, *n* = 80)_ = 10.59, *p* = 0.001; *χ*²_(1, *n* = 80)_ = 33.38, *p* < 0.001) for *N. girellae*, and the diurnal and nocturnal challenges for *Z. arugamensis* (*χ*²_(1, *n* = 60)_ = 93.65, *p* < 0.001; *χ*²_(1, *n* = 20)_ = 60.24, *p* < 0.001). However, there was no treatment effect for the diurnal *C. irritans* parasitic stage (on fish: *χ*²_(1, *n* = 80)_ = 0.16, *p* = 0.68) model, suggesting that none of the shrimp species significantly reduced the parasitic stage of *C. irritans* during the day (Fig. 1a). Therefore, only models with a treatment effect were considered for further pairwise comparisons between treatment and control groups per shrimp species.

### Cryptocaryon irritans

All shrimp species reduced *C. irritans* trophonts on infected fish nocturnally only (Fig. 1b). *Lysmata vittata* reduced trophonts by ~31.7% (*χ*²_(1, *n* = 20)_ = 11.51, *p* < 0.001), followed closely by *L. amboinensis* and *U. antonbruunii* with a reduction of ~28.7% and 23.4% (*χ*²_(1, *n* = 20)_ = 11.56, *p* < 0.001; *χ*²_(1, *n* = 20)_ = 48.59, *p* < 0.001), respectively. *Stenopus hispidus* reduced trophonts by ~11.5% (*χ*²_(1, *n* = 20)_ = 11.4, *p* < 0.001).

All shrimp reduced tomonts (non-parasitic stage) both diurnally and nocturnally, with the exception of *S. hispidus*, which only reduced tomonts diurnally (Fig. 1c,d). However, shrimp species did not perform equally, and some showed preference for either diurnal or nocturnal performance (Fig. 1c,d). *Lysmata vittata* out-performed all other shrimp species, reducing the tomonts by ~69.4% diurnally (*χ*²_(1, *n* = 20)_ = 23.07, *p* < 0.001; Fig. 1c) and 97.9% nocturnally (*χ*²_(1, *n* = 20)_ = 837.73, *p* < 0.001; Fig. 1d). *Lysmata amboinensis* reduced tomonts ~48.7% diurnally (*χ*²_(1, *n* = 20)_ = 18.93, *p* < 0.001; Fig. 1c) and 75.3% nocturnally (*χ*²_(1, *n* = 20)_ = 35.92, *p* < 0.001; Fig. 1d), while *U. antonbruunii* reduced tomonts diurnally by ~20.8% (*χ*²_(1, *n* = 20)_ = 5.13, *p* = 0.02; Fig. 1c) and 46.8% nocturnally (*χ*²_(1, *n* = 20)_ = 15.04, *p* < 0.001; Fig. 1d). *Stenopus hispidus* reduced tomonts diurnally by ~17.5% (*χ*²_(1, *n* = 20)_ = 8.14, *p* = 0.004; Fig. 1c).

### Neobenedenia girellae

Only *Lysmata amboinensis* and *U. antonbruunii* reduced *N. girellae* on infected fish both diurnally and nocturnally, while both *L. vittata* and *S. hispidus* only performed nocturnally. *Lysmata amboinensis* reduced infection by ~32% diurnally (*χ*²_(1, *n* = 20)_ = 7.25, *p* = 0.007; Fig. 2a) and 34.6% nocturnally (*χ*²_(1, *n* = 20)_ = 13.37, *p* < 0.001; Fig. 2b), followed closely by *U. antonbruunii* with diurnal, and nocturnal reduction of ~30% and 29.3% (*χ*²_(1, *n* = 20)_ = 6.81, *p* = 0.009; *χ*²_(1, *n* = 20)_ = 10.92, *p* < 0.001), respectively (Fig. 2a,b). *Lysmata vittata* and *S. hispidus* reduced infection nocturnally by ~23.6%, and 23%, (*χ*²_(1, *n* = 20)_ = 4.18, *p* = 0.04; *χ*²_(1, *n* = 20)_ = 8.21, *p* = 0.004), respectively (Fig. 2a,b).

Both *Lysmata* species and *S. hispidus* reduced *N. girellae* egg numbers both diurnally and nocturnally (Fig. 2c,d). However, *U. antonbruunii* did not reduce egg numbers at all (Fig. 2c,d). *Lysmata vittata* reduced egg numbers by ~74.4% and 86.1% (*χ*²_(1, *n* = 20)_ = 36.17, *p* < 0.001; *χ*²_(1, *n* = 20)_ = 50.64, *p* < 0.001), diurnally and nocturnally, respectively (Fig. 2c,d). This was followed by *L. amboinensis*, which reduced egg numbers by ~49% diurnally (*χ*²_(1, *n* = 20)_ = 13.05, *p* < 0.001; Fig. 2c) and 79% nocturnally (*χ*²_(1, *n* = 20)_ = 27.36, *p* < 0.001; Fig. 2d). *Stenopus hispidus* reduced eggs by ~35.9% diurnally and 28.8% nocturnally (*χ*²_(1, *n* = 20)_ = 9.3, *p* = 0.002; *χ*²_(1, *n* = 20)_ = 6.54, *p* = 0.01), respectively (Fig. 2c,d).

### Zeylanicobdella arugamensis

**Figure 4:**
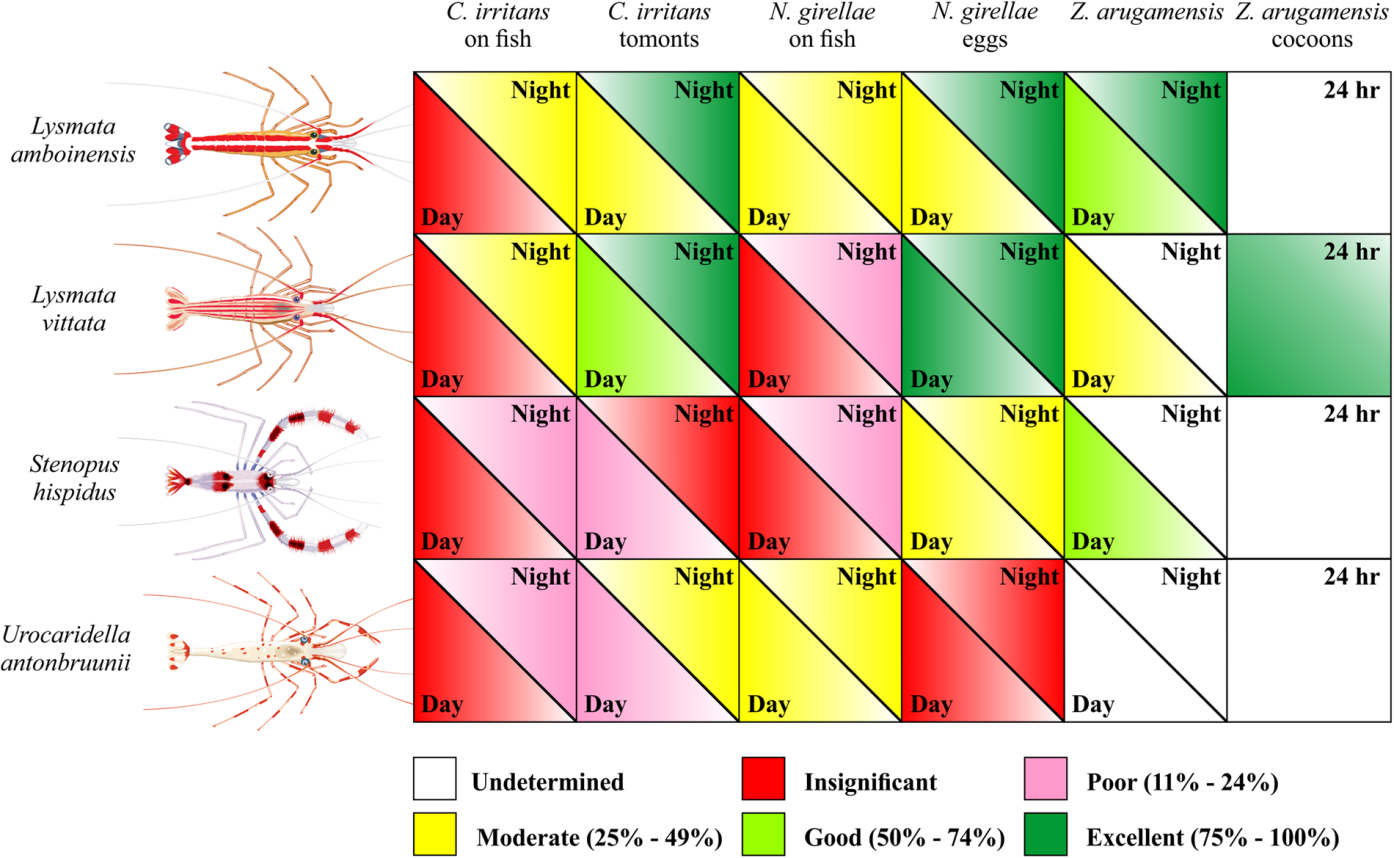
Shrimp species performance matrix. Ranking: Undetermined, no current data; Insignificant, *p* > 0.05; Poor, 11–24% reduction; Moderate, 25–49% reduction; Good, 50–74% reduction; Excellent, 75–100% reduction.

Some leeches vacated the host fish during experimentation and were recovered from the sides of the treatment and control tanks. Analyses were performed on the combined numbers of leeches recovered from the fish and tank surfaces to provide a result for overall leech reduction by shrimp (Fig. 3a). *Lysmata amboinensis* reduced leeches by ~65% diurnally (*χ*²_(1, *n* = 20)_ = 29.81, *p* < 0.001; Fig. 3a), and by ~77% nocturnally (*χ*²_(1, *n* = 20)_ = 60.24, *p* < 0.001; Fig. 3a); *S. hispidus* reduced leeches on fish diurnally by ~74% (*χ*²_(1, *n* = 20)_ = 39.44, *p* < 0.001; Fig. 3a); *L. vittata* reduced numbers of leeches by ~25% (*χ*²_(1, *n* = 20)_ = 19.4, *p* < 0.001; Fig. 3a), and leech cocoons over 24 hours by ~97% (*χ*²_(1, *n* = 20)_ = 265.95, *p* < 0.001; Fig. 3b) (see Supplemental File S1). A performance matrix was constructed based on the performance of each shrimp species, against all parasite species (Fig. 4). It indicated that *L. vittata* out-performed all other shrimp species in consuming parasites’ environmental stages.

## Discussion

Parasites in high numbers can cause mortalities in fish populations. Visible parasites, and the physical damage caused by them, can result in direct rejection by consumers, which can be costly to the aquaculture farmer, wholesaler and retailer^31^. These problems are often mitigated at farm-level through targeted applications of chemical therapies^6^. These practices contribute additional negative consumer sentiment^2,3^. In response to this, appropriate biocontrols could reduce both the frequency of chemical interventions, and the parasites that result in unsightly damage.

Our study is the first to demonstrate the potential of four cleaner shrimp species as biocontrols against three economically important fish parasite species in aquaculture, and supports peppermint shrimp *Lysmata vittata* as the first candidate species for further testing under aquaculture conditions. Cleaner shrimp may offer superior benefits to traditionally-used cleaner fishes as biocontrols as they are also capable of reducing parasite reinfection pressure directly by consuming environmental life-stages which are resistant to chemical therapies^30^. Employing these shrimp to reduce environmental life-stages also implies that no direct contact between shrimp and client fish is needed, and therefore shrimp predation risk is minimised. Additionally, shrimp are not susceptible to the ectoparasites of fishes they would be employed to control, which has become a recent concern with cleaner fishes used in the Atlantic salmon industry^23,32–35^.

An arguable limitation to using cleaner shrimp is their availability and cost, e.g. *L. amboinensis*. However, recent technological advances have supported successful captive breeding of cleaner shrimp species^36^. *Lysmata vittata* is currently produced commercially in Australia for the ornamental trade, and the cost per unit is based on the scale of demand. This shrimp has a large geographic distribution in the Asia-Pacific region, and through parts of the Indo-Pacific and North to Russian waters^37^. This distribution range incorporates some of the major sub-tropical and tropical aquaculture-producing nations^15^, including China, Philippines, Japan, Indonesia, and Australia^37^. Using *L. vittata* as a biocontrol in aquaculture offers a sustainable alternative to chemical interventions to treat fish ectoparasites, and may improve the overall sentiment of consumers regarding farmed fish.

We were able to test the performance of cleaner shrimp by maintaining three ectoparasite cultures in the laboratory. Our results show that different cleaner shrimp species vary in their cleaning performance. All four shrimp species tested in our study reduced the ciliate and monogenean on the fish directly (Figs 1a,b, 2a,b and 4). However, both *L. vittata* and *S. hispidus* were strictly nocturnal when removing these parasite species off the fish (Figs 1b, 2b and 4), while both *L. amboinensis* and *U. antonbruunii* reduced monogeneans on fish diurnally and nocturnally (Figs 2a,b and 4). None of the four shrimp species reduced ciliates on the fish diurnally (Figs 1a and 4). This may simply indicate a shrimp preference for nocturnal removal of this parasite only, a difference in the host behaviour, or behavioural changes of the parasite itself on the fish, making it more susceptible to predation nocturnally. Our experiment used 2 day old trophonts at 24 °C, and we did not recover any protomonts or tomonts in the experimental tanks. This suggests that trophonts were not yet vacating the host in our experiment. Some leeches did vacate the host during the challenges. We tried to avoid this by specifically selecting sub-adult leeches, but this behaviour likely represents a normal strategy after leeches have become satiated with a blood meal^38^. The numbers of leeches were limited so we completed diurnal and nocturnal challenges for one shrimp species (*L. amboinensis*), diurnal challenges for two (*L. vittata* and *S. hispidus*), and a 24 hour environmental stage (cocoons) challenge for *L. vittata* only (Fig. 3). *Lysmata amboinensis* reduced leeches both diurnally and nocturnally, while *L. vittata* and *S. hispidus* reduced leeches diurnally (Figs 3a and 4).

Not all shrimp reduced the environmental stage of ciliates and monogeneans (Figs 1c,d, 2c,d and 4). *Urocaridella antonbruunii* did not reduce the number of monogenean eggs either diurnally or nocturnally (Figs 2c,d and 4), suggesting that this shrimp species does not consume monogenean eggs. However, *U. antonbruunii* did reduce ciliate tomonts in the environment, and showed a better nocturnal than diurnal performance (Figs 1c,d and 4). Why this shrimp species would consume *C. irritans* tomonts and not monogenean eggs may be a consequence of their inability to masticate the latter, which have a hard protective sclerotin shell. Therefore, unlike the other three shrimp species, *U. antonbruunii* may be predisposed to consuming softer prey items. *Stenopus hispidus* reduced monogenean eggs diurnally and nocturnally (Figs 2c,d and 4), but was the only shrimp species that did not consume ciliate tomonts nocturnally (Figs 1d and 4). Both *Lysmata* species demonstrated the highest level of environmental stage reduction for ciliates and monogeneans, with an increased performance nocturnally (Figs 1c,d, 2c,d and 4). *Lysmata vittata* reduced leech cocoons over the 24 hour period (Figs 3b and 4). Overall, it was the best performer of parasite environmental stage reduction (Figs 1c,d, 2c,d and 4).

It was evident from the results of our environmental stage challenges that individual shrimp of the same species did not perform equally. The same phenomenon was previously observed for *L. amboinensis*^30^. Similarly *S. hispidus* was discussed in terms of different individual responses to environmental cues^39^. This phenomenon likely reflects shrimp foraging in an area-restricted search pattern, where the shrimp consume as much as they can on each chance encounter with a prey item. Historically, the legitimacy of *S. hispidus* as a cleaner shrimp had been questioned^26,29^. In the first laboratory-based empirical study on cleaner shrimp, *S. hispidus* did not remove the parasitic isopod *Anilocra haemuli* off host fish^26^. However, in a semi-natural microcosm, *S*. *hispidus* did appear to have a preference for consuming larger individual *Neobenedenia melleni* monogeneans, although it did not reduce parasite numbers on hosts^29^. Our data indicate that this shrimp species does significantly reduce ciliates, monogeneans and leeches on host fish. Apparent difference in the capacity of *S. hispidus* to reduce the closely related monogeneans *N. girellae* in our study and *N. melleni* in the previous macrocosm study^29^, is unlikely due to parasite species differences. Fish in the macrocosm were subjected to incoming water from an exhibit with a known infection of *N. melleni* continuously for at least 14 days^29^, and would therefore have been repeatedly infected naturally over this period. From our data, *S. hispidus* demonstrated a nocturnal bias towards removal of ciliates and monogeneans off host fish. This supports the earlier conclusions, that *S. hispidus* is primarily nocturnal^29,40–42^. However, *S. hispidus* does clean fishes and turtles diurnally^43^, a behaviour which our results also support for the reduction of leeches. Diurnal cleaning by *S. hispidus* was suggested to be a function of changing light conditions^39^, but it appears that *S. hispidus* may prefer to prey on different fish ectoparasites diurnally or nocturnally, which may reflect more the behaviour of the clientele that have these parasites.

*Urocaridella antonbruunii* is the first *Urocaridella* species reported to reduce monogeneans. Although a similar study found that *Urocaridella* sp. c (yellow-beaked cleaner shrimp) eat dead *Benedenia* sp. monogeneans offered to them, only *Ancylomenes holthuisi* was used to test shrimp efficacy at removing this monogenean on *Ctenochaetus striatus*^27^. The same shrimp, *U*. sp. c, was also used to evaluate hunger levels on cleaning time, using *Cephalopholis cyanostigma* infected with *Benedenia* sp. monogeneans^44^. Therefore *U*. sp. c likely also consumes monogeneans, at least of the family Capsalidae. Currently, temperate and tropical cleaner shrimp that eat monogeneans represent the families Hippolytidae, Palaemonidae, and Stenopodidae.

Cleaner shrimp may provide a different type of cleaning service than that of cleaner fishes^45^. This is supported by an apparent ‘cleaning structure discordance’, evident by an apparent lack of competition between cleaner fish and shrimp observed in the wild, and a difference in temporal interactions with the same clients^45^. The specific cleaning function of shrimp remains largely unresolved, driven by underexplored proximate causes^44^. Our data suggest that many cleaner shrimp may likely offer the combined benefit of a symbiotic cleaning service, and non-symbiotic cleaning as a by-product benefit in the shelters shared by their resting clients, which probably reduces parasite reinfection success. Parasites have different life cycle and reproductive strategies. For example, some monogeneans release eggs specifically following the onset of darkness^46^ or produce more eggs at night^47^, and the ciliate *C. irritans* vacates the host at night. Many parasites have infective stages that also emerge nocturnally, e.g. *C. irritans*^48^. Cleaner shrimp appear to actively reduce parasites nocturnally, and may therefore be an important source of parasite control on a reef at night when diurnal fish cleaners, like *Labroides dimidiatus*, are inactive.

Further research into the potential use of cleaner shrimp in aquaculture is warranted. A recent study estimated the global diversity of cleaner shrimp species to be approximately 51 known species from 11 genera, representing six families^49^. These taxa include tropical, sub-tropical and temperate marine representatives. Further work could explore whether client fish species recognise cleaner shrimp species as cleaners under aquaculture conditions to determine whether direct cohabitation, as is used in salmon farming with cleaner fishes, is feasible with shrimp. However, we have demonstrated that cohabitation is not necessary in reducing parasite problems. The advantages of exploiting shrimps’ natural predatory behaviour of parasites and other pathogens, particularly their environmental stages, implies a wealth of potentially new solutions to existing and future aquaculture health problems.

## Methods

### Animal ethics

Ethics approval was granted under the James Cook University Ethics Committee Permit numbers A2260, and A2457, conforming strictly to the national regulations set out in the National Health and Medical Research Council (2013) Australian code for the care and use of animals for scientific purposes, 8th edition, under Section 39 of the National Health and Medical Research Council Act, 1992. *Neobenedenia girellae* and *Z. arugamensis* could be recovered from fish without destructive sampling. Therefore, all fish used in these challenge experiments were returned to their original holding area after experimentation. *Cryptocaryon irritans* is a subcutaneous fish parasite but also infests the gill tissue. For accurate counts on fish it was therefore necessary to humanely euthanase all fish in the *C. irritans* challenges using an anaesthetic overdose of 2-Phenoxyethanol at 1.5 ml/L for 10 minutes^50^ prior to sampling.

### Animal acquisition, maintenance and biosecurity

Thirty individuals each of the cleaner shrimp *L. amboinensis*, *L. vittata*, *S. hispidus*, were purchased from a commercial, Australian ornamental fish/invertebrate supplier (Cairns Marine) and 30 *U. antonbruunii* individuals were collected using SCUBA off Lizard Island, Queensland, Australia. All fish used to culture parasitic organisms, or used directly in the experiment, were obtained from commercial hatcheries in Queensland, Australia. All fish were given a 5-minute freshwater bath on arrival in the laboratory to remove potential ectoparasites. The fish were then quarantined in five 70 L plastic aquaria coupled to a recirculating marine life-support system for 30 days before being transferred to a single 5000 L tank prior to the experiment. All cleaner shrimp were housed separately in individual 3 L plastic aquaria connected to a separate recirculating marine life-support system. Prior to experimentation, fish were fed daily with 3 mm juvenile marine fish floating pellets (Ridley Agriproducts Pty Ltd; product code: 107578) and shrimp were given defrosted *Mysis* sp. shrimp daily, to satiation. During the experiments, shrimp were fed after removal from experimental tanks. To avoid the potential for cross-contamination, and to allow for effective biosecurity, each parasite-fish challenge experiment was run separately for each shrimp species, and equipment disinfected with 50 mg/L sodium hypochlorite between trials following OIE guidelines. All effluent water from experimentation was first disinfected with sodium hypochlorite at the same final concentration prior to disposal via sewer system.

### Ectoparasite source and monoculture methods

All ectoparasites were obtained from species monocultures established in the Marine Parasitology Laboratory at James Cook University, Townsville, Australia. *Cryptocaryon irritans* was cultured continuously in the laboratory at 26 °C and 35 ppt on individual juvenile *L. calcarifer* (~15 cm total length). Freshwater juvenile *L. calcarifer* were acclimatised to seawater over two days before being introduced directly into the parasite culture tank (60 L). These fish were then allowed to support a low intensity, single life cycle of *C. irritans*. Once visible trophonts had vacated the host, the fish were removed from the culture, and re-acclimatised to freshwater (which kills any remaining parasites) before being returned to their freshwater aquarium. To boost numbers of *C. irritans* prior to experimentation, additional fish were added to the culture to increase tomont production for harvesting from the culture tank. Individual *L. calcarifer* were also used to maintain a continuous monoculture of *N. girellae* in the laboratory, following previously published methodology^51^. *Zeylanicobdella arugamensis* was cultured continuously at 30 °C and 28 ppt sequentially on a cohort of 40 individual *Epinephelus lanceolatus* with an approximate average individual mass of 1.5 kg each (i.e. one life-cycle completion per individual, rotated through the culture). Adult leeches vacate the host to produce cocoons which they deposit directly onto the glass surfaces of the culture tank. Once adult leeches had begun producing cocoons, the existing host was removed from the culture, and replaced with a new host fish coinciding with the emergence of juvenile leeches.

### Experimental design

Diurnal and nocturnal challenges were performed for the parasitic and the environmental stages of *C. irritans* and *N. girellae* for all shrimp species. The numbers of leeches were limited so we completed diurnal and nocturnal challenges for *L. amboinensis*, and diurnal challenges for *L. vittata* and *S. hispidus* only (i.e. excluding *U. antonbruunii*). The ability of only *L. vittata* to reduce leech cocoons was evaluated.

**Figure 5:**
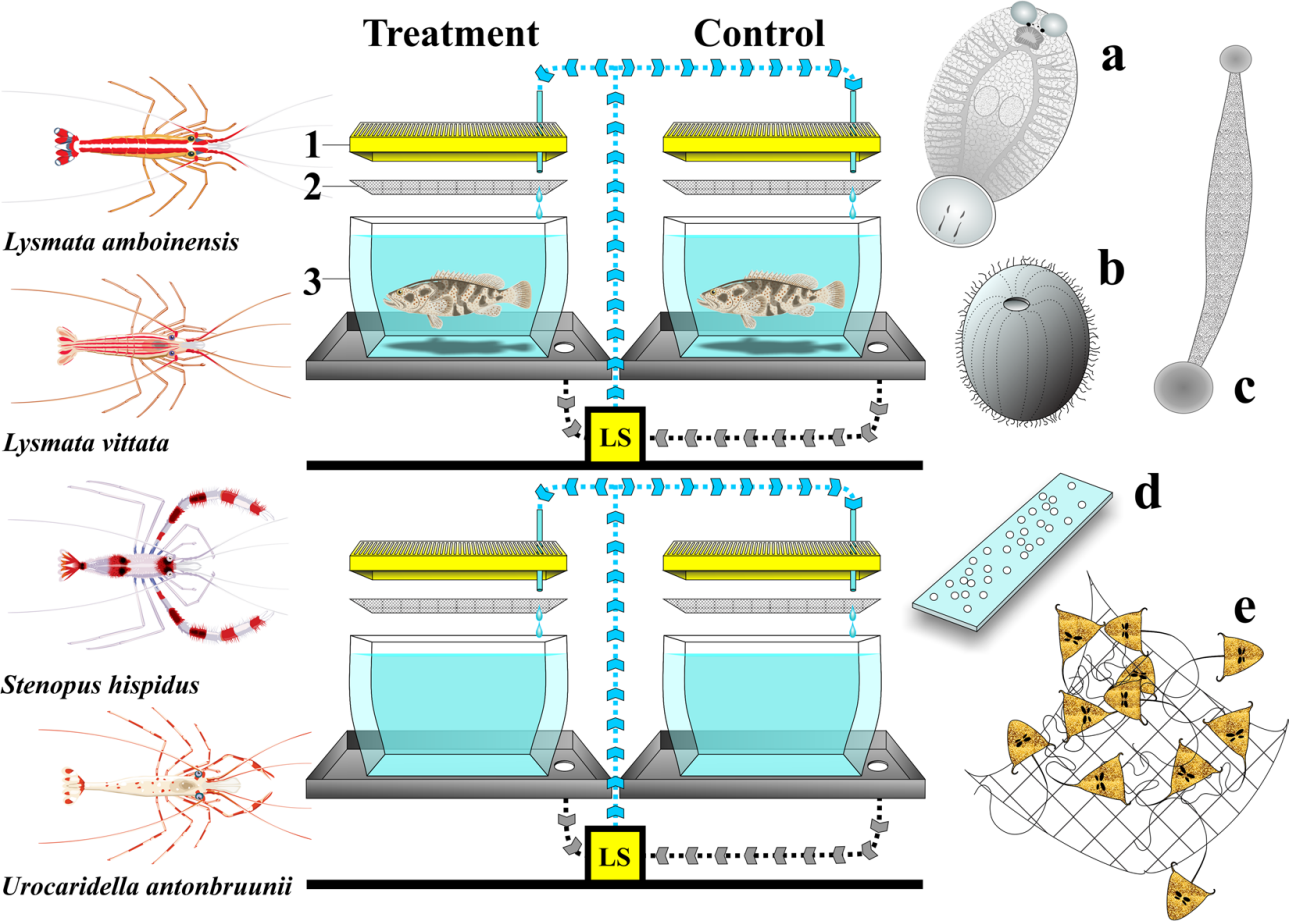
Experimental design demonstrating treatment and control setup for a single replicate for parasitic (on fish) and environmental stages, and shrimp species used. (**a**) *Neobenedenia girellae*. (**b**) *Cryptocaryon irritans* trophont. (**c**) *Zeylanicobdella arugamensis*. (**d**) *Cryptocaryon irritans* tomonts (cysts) or *Z. arugamensis* cocoons on microscope slide. (**e**) Embryonated *Neobenedenia girellae* eggs attached to bridal tulle. LS. Recirculating seawater life-support system with filtered influent (blue arrows), and effluent (grey arrows) schematic water flow; 1. Fitted tank lid; 2. 60 µm mesh tank cover; 3. Individual experimental tank positioned inside a water catchment tray.

A total of four hundred individual farmed juvenile *E. coioides* (~15 cm in total length) were used for the parasitic stage challenges with *N. girellae*, *C. irritans*, and *Z. arugamensis* (i.e. 10 individual treatment and 10 individual control fish per challenge, for each parasite species), per shrimp species used (Fig. 5). The shrimp and client fish species were selected because they share an overlapping distribution in Australia and through parts of the Asia-Pacific, and the client is a valued aquaculture species in this region. For each parasitic stage challenge, infected fish were randomly assigned to 20 identical 3 L plastic aquaria (10 treatment and 10 control aquaria), coupled to a recirculating marine life-support system to maintain water quality conditions over the experimental period (24 °C and 35 ppt; Fig. 5). Random numbers for fish assignment were generated using a random number generator.

For each parasitic stage challenge, 10 individual shrimp were cohabited with the 10 treatment fish only, and the experiment ran for 12 hours (either 12 hours diurnal; 07:00-19:00, or 12 hours nocturnal; 19:00-07:00, in an artificially light-controlled laboratory). All tanks were fitted with a tightly fitting 60 µm mesh cover secured over the water surface area by the fitted lid of each tank to allow water exchange without any potential parasite loss (Fig. 5).

After 12 hours, all fish were removed from their treatment and control tanks. Fish infected with *N. girellae* were placed into individual glass beakers containing dechlorinated freshwater and an anaesthetic concentration of 2-Phenoxyethanol at 0.15 ml/L^52,53^ for 5 minutes to anaesthetise the fish, and to kill and to dislodge *N. girellae*. Similarly, fish with leeches were placed into individual glass beakers, but containing a seawater-anaesthetic concentration of 2-Phenoxyethanol to immobilise the fish for inspection. These fish were moved individually to a large glass petri dish and observed at 20X magnification under a Leica M60 dissection microscope. Fish were kept submerged in the same seawater-anaesthetic solution while carefully removing any remaining leeches with forceps. All anaesthetised fish were returned to their holding aquarium to recover. The contents of each beaker and each 3 L tank was filtered through a 60 µm sieve and transferred separately to a glass petri dish for counting of parasites under the dissection microscope. The 60 µm mesh tank covers and tank sides were also inspected for any dislodged parasites. All *N. girellae* and *Z. arugamensis* were counted and preserved in vials of 70% ethanol.

Fish infected with *C. irritans* were placed into individual glass beakers containing seawater and a euthanasia concentration of 2-Phenoxyethanol at 1.5 ml/L for 10 minutes^50^. Thereafter, each fish was placed into a shallow glass Petri dish and *C. irritans* cells counted on the entire body surface under the dissection microscope at 20 X magnification. After counts of the body surface were made, all gill arches were excised to facilitate separate observations and counts. The seawater solution in each beaker, and experimental tank, and each 60 µm tank cover was similarly scrutinised for any dislodged *C. irritans* cells. All *C. irritans* cells were recorded per fish, and each individual fish and their gills was fixed in 10% phosphate-buffered formalin.

The same experimental setup was used for the environmental stage challenges excluding fish (Fig. 5.) We used 10 treatment and 10 handling control tanks per challenge. An average of 50 (30–88) *Neobenedenia girellae* eggs, 56 (30–131) *C. irritans* tomonts (cysts), or 88 (27–221) *Z. arugamensis* cocoons were introduced to both the treatment and the handling control tanks. These challenges ran for 12 hours diurnally or nocturnally as for the parasitic stage challenges, with the exception that the leech cocoon trial was completed over 24 hours, combining 12 simulated diurnal and nocturnal hours. At the end of each trial, the *N. girellae* eggs, *C. irritans* tomonts, or *Z. arugamensis* cocoons were counted under the dissection microscope.

### Infection of fish with parasites

*Cryptocaryon irritans* infects the skin and gill tissue of its host fish. Adult trophont cells vacate the host and encyst (tomont stage) on the substrate in the environment where they undergo cell division to produce hundreds of reinfective, free-swimming microscopic theronts^54^. Tomont “carpets” (cell aggregations forming a tomont monolayer)^54^ were collected from the glass substrate of the culture tank with a scalpel blade. These were washed in fresh seawater and incubated at 24 °C and 35 ppt in a glass petri dish to ensure mass emergence of theronts for experimental infection using an artificial 12 hour day/night light regime. Theronts (~4 hours post-emergence)^55^ were then transferred to a beaker of fresh, filtered seawater (24 °C and 35 ppt) via pipette and evenly distributed by continuous gentle aeration. Samples of the solution were taken with a pipette and observed on a haemocytometer. The theront-seawater solution was serially diluted using additional seawater to obtain a concentrated solution of 1000 cells per ml, following subsequent haemocytometer sample observations as required.

All fish for the *C. irritans* challenge experiments were placed into identical non-recirculating 3 L plastic aquaria containing gently aerated, fresh, filtered seawater (24 °C and 35 ppt), and 1 ml of the theront-seawater solution was added via pipette to a final concentration of ~166 theronts/L. This concentration was used because theronts demonstrate a relatively low invasion success rate of between 5% and 20%^48^. Host fish and theronts were cohabited for 1 hour to allow successful infection^54^, cf.^55,56^. Thereafter all individual fish were removed to identical non-recirculating 15 L plastic tubs containing gently aerated, fresh, filtered, seawater (24 °C and 35 ppt) for 48 hours to allow trophonts to develop but not to mature^54^. Each fish was given a complete seawater exchange daily at the same temperature and salinity to maintain water quality. After 48 hours post-infection, individual fish were removed and used for experimentation.

For the *N. girellae* challenge experiments, embryonated eggs recovered from the culture were placed into a glass Petri dish containing fresh, filtered seawater, and hatched at room temperature^57^. Thirty freshly hatched (<2 hours post-hatch)^30^ free-swimming larvae (oncomiracidia) were removed via pipette and introduced to each identical non-recirculating 15 L plastic tub containing an individual fish, and gently aerated in fresh, filtered seawater (24 °C and 35 ppt). Larval *N. girellae* were established and grown on fish for 144 hours prior to experimental use to avoid parasites reaching sexual maturity^57^, but giving them enough time to grow to a size to facilitate easy observation and recovery. This was done to avoid any eggs being produced in the experimental system that could potentially hatch and reinfect fish. Over this period, each fish was given a complete seawater exchange daily at the same temperature and salinity to maintain water quality.

Leeches were collected by anaesthetising the host fish with 0.15 ml/L 2-Phenoxyethanol and careful removal with a wet cloth. Only sub-adult leeches were used for experimentation. Fish were introduced to individual glass beakers of seawater containing 10 leeches each. On contact, leeches attached immediately, and fish were carefully transferred thereafter to the experimental or control tanks.

### Preparation of environmental stages

*Neobenedenia girellae* produces large numbers of straw-coloured tetrahedral eggs which contain a tendril which can entangle on environmental structures. Egg embryonation can be observed by the development of pigmented eyespots in each developing oncomiracidium within the egg^51^ (see Fig. 5). Individual groups of embryonated eggs entangled on a ~1 cm² piece of fine bridal tulle (Fig. 5) were glued to a small glass petri dish for stability, prior to random allocation in the experiment.

Glass microscope slides were placed over the benthic surface area of both the *C. irritans* and *Z. arugamensis* culture tanks, allowing tomonts to encyst and therefore to attach, or cocoons to be cemented dorsally to one side, over 24 hours (Fig. 5). These slides were removed to the dissection microscope and the number of tomonts or cocoons counted and recorded in pencil on each slide, prior to random allocation in the experiment.

### Statistical analyses

All analyses were performed in R (Version 3.4.0; R Development Core Team 2017)^58^, using the packages ‘glm2’^59^ for generalised linear models, and ‘car’^60^. Count proportions were used as the response variable for all analyses, i.e. the recovered proportion of the originally introduced amount of the parasitic (on fish) and the environmental stage of both parasite species to each of the treatment, and handling control tanks. Two approaches were used to explore the data for each parasite species, and data for each diurnal and nocturnal parasite experiment was run separately. First, we ran generalised linear models with a binomial regression and “logit” link with ‘*shrimp species*’ and ‘*treatment*’ (shrimp presence vs shrimp absence), and the interaction ‘*shrimp* × *treatment*’ as potential explanatory variables. These models indicated overdispersion. Therefore we re-ran the analyses with a quasibinomial regression and “logit” link to account for overdispersion. This was done to detect whether an effect of treatment was significant, or not, in each model. Based on these results we explored pairwise comparisons for each separate shrimp species using ‘*treatment*’ as the explanatory variable. We ran each of these as separate generalised linear models with a quasibinomial regression and “logit” link, analysed with Anova() in the ‘car’ package.

## Electronic supplementary material

Supplementary Information

## Data Availability

All data from which analyses were performed are available at, 10.4225/28/5b343a73f35f7.
